# A Case of Dedifferentiated Laryngeal Liposarcoma With Metachronous Transformation Into a Neoplasm With Myxofibrosarcomatous Elements

**DOI:** 10.7759/cureus.30901

**Published:** 2022-10-31

**Authors:** Paraskevi Karamitsou, James Philip Skliris, Aikaterini Karamitsou, Evropi Forozidou, Alexandros Poutoglidis

**Affiliations:** 1 Otorhinolaryngology, 'G. Papanikolaou' General Hospital, Thessaloniki, GRC; 2 Pathology, 'G. Papanikolaou’ General Hospital, Thessaloniki, GRC; 3 Surgery, ‘G. Papanikolaou’ General Hospital, Thessaloniki, GRC

**Keywords:** total laryngectomy, liposarcoma, head and neck, tumor, oncology

## Abstract

Liposarcomas are rare mesenchymal tissue tumors and are divided into subtypes based on their histopathological characteristics. They are mostly well-differentiated neoplasms with the tendency to recur locally. Lymph node involvement or distant metastases have been reported as extremely rare. Common manifestations are progressive dyspnea, dysphagia, choking, and stridor. Surgical excision of laryngeal liposarcomas is considered the gold standard treatment modality for disease eradication. In persistent or recurrent cases, a total laryngectomy should be performed. There is much controversy regarding the role of radiotherapy which is mostly used as adjuvant treatment in specific cases. We present a case of dedifferentiated laryngeal liposarcoma with multiple recurrences and metachronous transformation to a neoplasm with myxofibrosarcomatous elements.

## Introduction

Squamous cell carcinoma is the most common neoplasm of the larynx [1]. Non-squamous cell carcinomas are fairly rare tumors, constituting only 1% of all laryngeal malignancies [2]. Liposarcomas are tumors of mesenchymal tissue origin and are divided into five subtypes based on their histopathological characteristics: a) well-differentiated, b) myxoid, c) round cell, d) undifferentiated, and e) pleomorphic [2, 3]. The vast majority of laryngeal liposarcomas are low-grade tumors, mainly associated with the well-differentiated subtype [4].

Surgical excision is considered the gold standard treatment modality [4]. Total laryngectomy may be unavoidable in large tumors or in cases with multiple recurrences. We present a case of a dedifferentiated supraglottic laryngeal liposarcoma with multiple recurrences and a metachronous transformation to a neoplasm with myxofibrosarcomatous elements.

## Case presentation

A 78-year-old, non-smoker male presented to the Otolaryngology Department with the chief complaint of a two-month history of progressive dyspnea. Hoarseness, difficulty in swallowing, and loss of appetite were also reported as accompanying symptoms. The patient’s medical history included ischemic heart disease and benign prostate hyperplasia. He was hemodynamically stable with mild inspiratory stridor.

**Figure 1:**
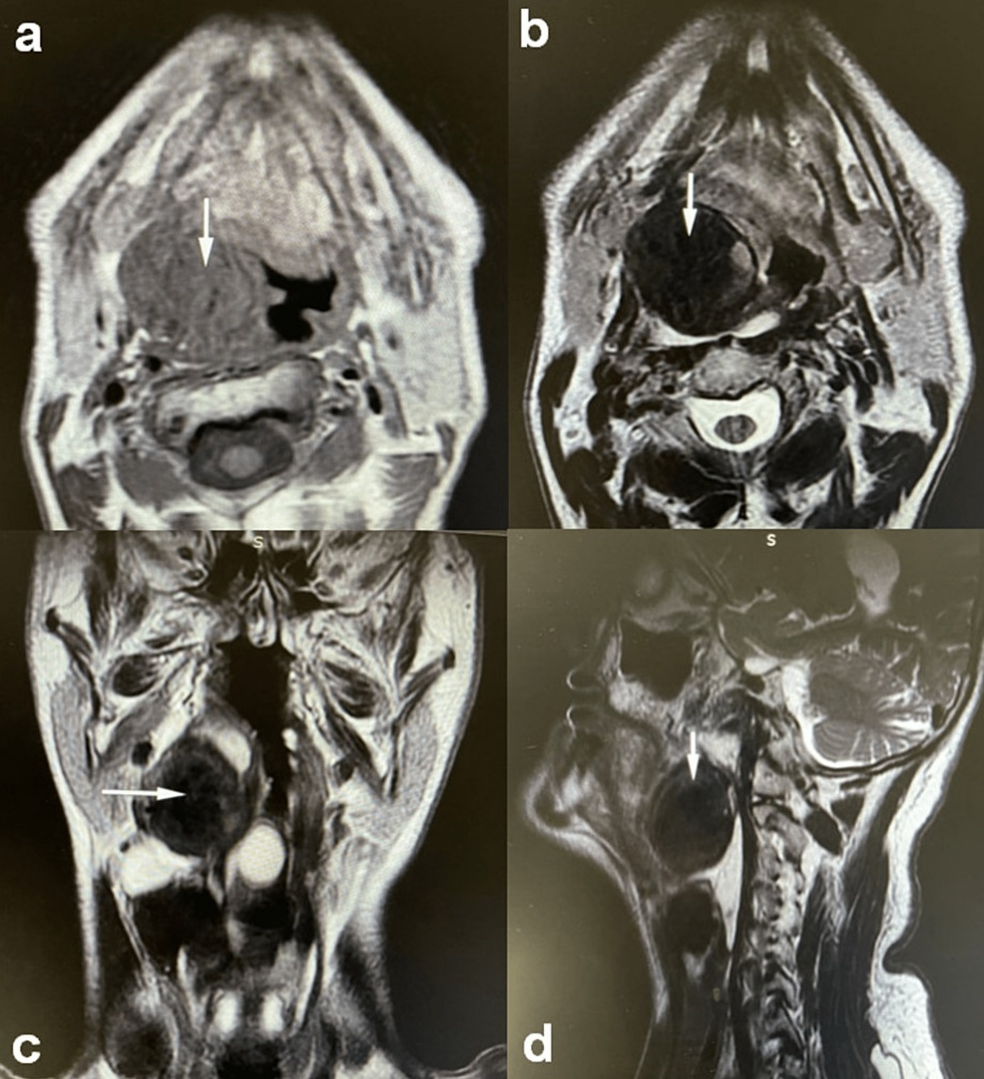
The MRI depiction of a supraglottic laryngeal mass MRI. A growth (white arrow) (5.3cm x 3.9cm) in the supraglottis with inhomogeneous paramagnetic contrast agent uptake and repulsion of adjacent anatomical structures. a. Axial (T1W1 sequence), b. Axial (T2W1 sequence), c. Coronal (T2W1 sequence), d. Sagittal (T2W1 sequence)

A thorough physical examination was performed. Inflexible laryngoscopy revealed a large supraglottic mass. The vocal cords were barely visible, without clear glottis involvement. A neck examination did not reveal palpable lymph nodes. Laboratory tests were normal. Magnetic Resonance Imaging (MRI) revealed a 5.3cm x 3.9cm mass in the supraglottic laryngeal region with inhomogeneous paramagnetic contrast agent uptake and repulsion of adjacent anatomical structures. No infiltration was noted (Figure 1). 

**Figure 2:**
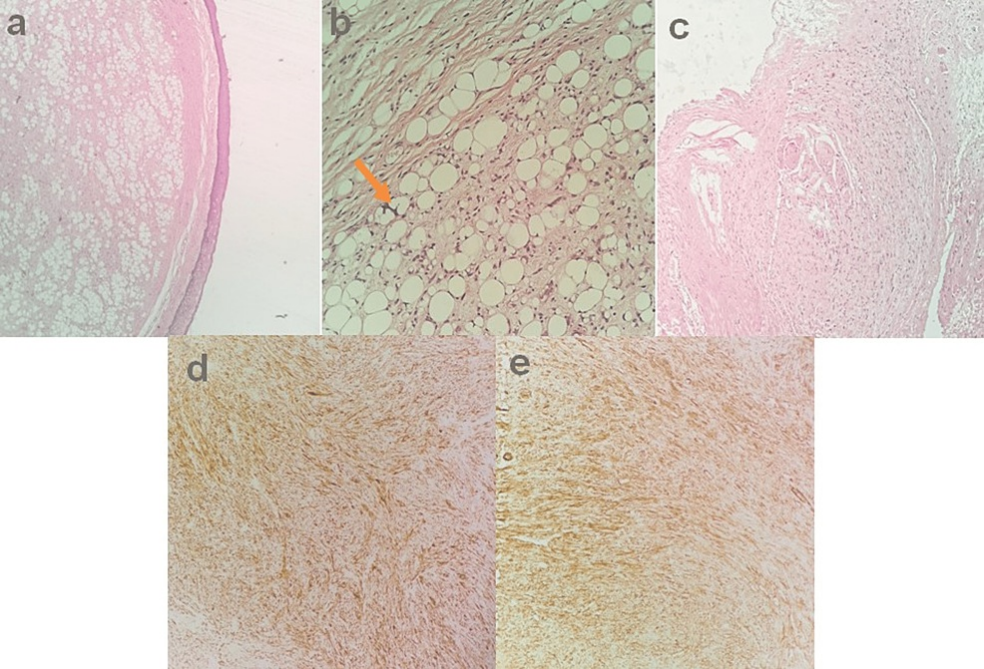
The histopathologic illustration of dedifferentiated liposarcoma a. Atypical lipomatous tumor/well-differentiated liposarcoma (alt/wdl) element in proximity with pharyngeal mucosa (Hematoxylin and Eosin staining (HE), 4X), b. Higher power magnification of an alt/wdl element of dedifferentiated liposarcoma. Lipoblasts (orange arrow) can be noticed (HE, 20X), c. Invasion of muscle fibers from the tumor (HE, 10X), d. Desmin positivity in non-lipogenic element (Immunohistochemistry (IHC), 10X), e. H-caldesmon positivity in non-lipogenic element (IHC, 10X)

A biopsy via direct microlaryngoscopy was performed. Histology indicated the presence of a malignant tumor with elements of an atypical lipomatous tumor/well-differentiated liposarcoma (alt/wdl) and restricted areas comprising a non-lipomatous sarcoma, establishing the diagnosis of dedifferentiated liposarcoma. Immunohistochemistry showed S-100 and CD34 positivity in alt/wdl constituents, while desmin and H-caldesmon stained the non-lipomatous sarcoma areas of the neoplasm (Figure 2). 

Subsequently, the tumor was extracted via a transoral approach, under general anesthesia and the histology report confirmed the diagnosis of dedifferentiated liposarcoma.

Two months later, the patient presented for his follow-up appointment and local recurrences were detected. A smooth outlined 3cm lump was firmly attached to the thyroid cartilage, and a second mass in the right arytenoid cartilage was found. Both masses were excised and concomitant histopathological examination reconfirmed the initial diagnosis of dedifferentiated liposarcoma.

**Figure 3:**
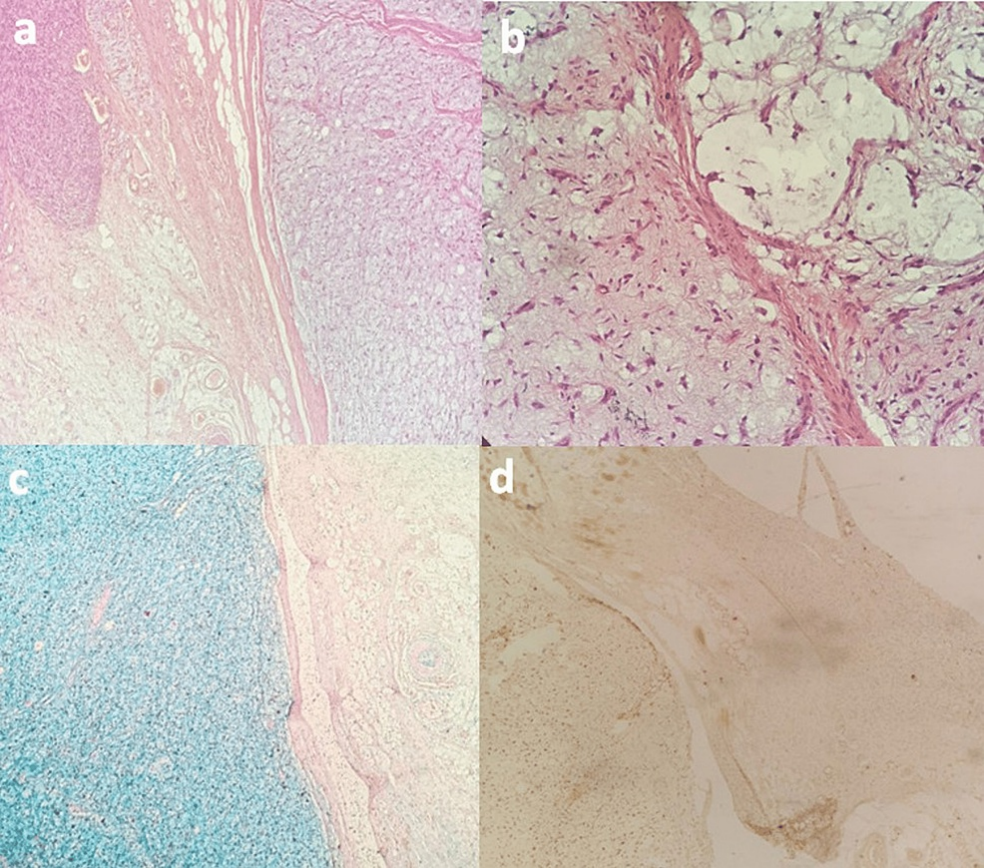
The myxofibrosarcomatous element of the neoplasm a. Myxofibrosarcomatous element obvious on the right side of the image (Hematoxylin and Eosin (HE), 4X), b. Myxofibrosarcomatous element: atypical cells in the myxoid background (HE, 20X), c. Alcian blue staining acid mucins of the myxofibrosarcomatous element of the tumor (Alcian blue histochemical stain, 4X), d. S-100 negativity in myxofibrosarcomatous element (immunohistochemistry (IHC), 10X)

Twelve months later, the patient presented again to our Emergency Department with stridor, dyspnea, and occasional choking episodes. Flexible nasolaryngoscopy revealed a huge mass occupying almost the whole larynx. A tracheotomy under local anesthesia was performed to secure the airway and the tumor was resected under direct microlaryngoscopy. The histology report described the presence of a foci with myxofibrosarcomatous elements among the sarcoma, evident in the alcian blue histochemical stain (Figure 3). 

Our hospital's multidisciplinary team (MDT) recommended adjuvant neck radiotherapy due to the tumor's persistence and histopathological characteristics. The patient received a total dose of 4200 cGy in 14 sessions. Twelve months after radiotherapy, the patient remained free of disease. He died eight months later due to a heart attack episode.

## Discussion

Liposarcomas are malignant mesenchymal tissue neoplasms, emerging from the adipose tissue and represent the second most common soft tissue sarcoma [2]. Less than 10% of liposarcomas appear in the head and neck area [2], while less than 1% arise from the larynx [3]. Atypical lipomatous tumor/well-differentiated liposarcoma and dedifferentiated liposarcoma comprise the largest subgroup of liposarcomas, mainly occurring in middle-aged or older patients [5].

Most of these cases involve the supraglottic larynx [4]. The main reported symptoms of laryngeal liposarcomas are progressive dyspnea, dysphagia, choking, and stridor [6]. Differential diagnosis includes benign lesions, such as lipomas, and other types of soft tissue or epithelial malignancies [4]. 

MRI is a helpful tool in establishing the diagnosis to distinguish liposarcomas from lipomas. However, imaging studies are not always adequate to determine the diagnosis [6]. Hemorrhage and necrosis in the MRI are in favor of liposarcomas, while complete fat suppression is indicative of lipomas [6]. Thus, histopathology remains the gold standard diagnostic tool [6].

Endoscopic resection should be performed only in limited cases, due to the recorded tendency of well-differentiated liposarcomas to recur at an extremely high rate [4, 7, 8]. The treatment of choice for laryngeal well-differentiated liposarcomas is wide surgical excision [4, 7]. In cases of tumors that threaten the airway, a tracheotomy should be initially performed to secure breathing. Total laryngectomy should be performed only in persistent cases, in multiple recurrences, and cases of a non-functional larynx [4]. Lymph node or distant metastases have been reported as extremely rare. Up to 10% of well-differentiated liposarcomas could dedifferentiate into dedifferentiated liposarcomas [5]. This transformation is associated with more aggressive clinical behavior [5].

Dedifferentiated liposarcomas have typically the appearance of undifferentiated pleomorphic or spindle cell sarcomas and arise de novo or in association with a preexisting well-differentiated liposarcoma [5]. In neoplasms that lack the component of a well-differentiated liposarcoma or in patients with no previous history of well-differentiated liposarcoma, the diagnosis of dedifferentiated liposarcoma might be quite difficult [5]. The role of radiotherapy in their management remains controversial [2, 9]. Adjuvant treatment with radiotherapy is usually recommended in cases of positive surgical margins, metastatic disease, or tumor transformation to a more aggressive sarcomatous subtype [7]. Myxoid subtypes seem to respond better to radiotherapy [7, 9]. The role of chemotherapy is still under investigation. According to some studies, chemotherapy should be individualized and might be useful in metastatic disease or as a palliative treatment [6]. 

## Conclusions

Head and neck liposarcomas constitute a rare clinical entity. They should be included in the differential diagnosis of laryngeal masses, especially in middle-aged or older patients. A timely diagnosis and treatment require high clinical suspicion. Wide surgical excision is reported to be the most appropriate treatment modality. More studies are required to clarify the role of radiotherapy and chemotherapy. To the best of our knowledge, this is the first case of dedifferentiated laryngeal liposarcoma with a metachronous transformation into a neoplasm with myxofibrosarcomatous elements. 
